# Correctional Services Canada Recruits: Their Mental Health During Training at Occupational Entry and the Role of Trauma Exposures in Prevalence of Mental Disorders and Suicide Behaviors

**DOI:** 10.1177/00938548261435837

**Published:** 2026-04-29

**Authors:** Arthi Chinna Meyyappan, Rosemary Ricciardelli, Sahar Dorniani

**Affiliations:** Memorial University of Newfoundland

**Keywords:** mental health, correctional officers, longitudinal study, PTSD, depression, trauma

## Abstract

Correctional officers (COs) in Canada experience a high prevalence of mental disorders, suicidal behaviors, and trauma exposure. However, recruits entering the Correctional Service of Canada tend to show lower prevalence than the general population, likely influenced by mandatory pre-employment mental health screening. We report self-report survey data from 792 CO recruits between 2018 and 2024, assessing mental health, suicidal behaviors, and trauma exposure before training. Recruits appeared generally mentally healthy: 7.2% screened positive for any disorder (1.6% posttraumatic stress disorder, 1.6% major depressive disorder, 1.8% GAD), and suicidal behaviors were rare (1.0% ideation, 0.2% attempts). Although most recruits reported multiple potentially traumatic events, specific high-impact trauma types, rather than cumulative trauma, were more strongly associated with positive screens. Recruits from certain occupational backgrounds showed elevated risk. Findings suggest robust baseline mental health despite trauma exposure and highlight the need for longitudinal research to understand how trauma type and preservice experiences shape mental health over time.

## Introduction

Correctional officer recruits (CORs) at Canada’s federal correctional system, Correctional Service of Canada (CSC), begin their careers after undergoing a mental health screening, which determines whether they continue in the recruitment process and proceed to training. Despite this screening, cross-sectional research has consistently demonstrated that correctional workers (CWs) experience a high prevalence of mental disorders both in Canada and internationally (Carleton et al., 2018; Fusco et al., 2021; Lavender & Todak, 2021; Ricciardelli et al., 2022). This study draws on data from more than 700 CORs, collected during their training and before their deployment in CSC penitentiaries. Given extensive evidence that COs are routinely exposed to potentially psychologically traumatic events (PTEs) throughout their careers (Konyk et al., 2021), we focus here on trauma exposure at occupational entry among recruits. We examine whether mental health outcomes are more strongly associated with cumulative trauma exposure, singular events, or repeated exposure to a unique type of trauma.

We begin this article with a review of the literature on the prevalence of mental disorders, suicide behaviors, and trauma exposure among CORs and correctional officers (COs), at occupational entry, followed by a discussion of cumulative versus noncumulative trauma. After outlining CSC’s recruitment screening process, we present our methods and results. The discussion considers the potential implications of screening practices and explores whether posttraumatic growth may help explain the low prevalence of mental disorders among CORs at baseline.

### Recruits and Mental Disorders

In a prior study, using a subset of the current sample (*n* = 265), scholars found only 4.9% of federal CORs screened positive for one or more current mental disorders, with 2.4% screening positive for posttraumatic stress disorder (PTSD) and 1.9% for major depressive disorder (MDD) (Easterbrook et al., 2022). The prevalence remains substantially lower than that observed among serving provincial and territorial COs, where 58% screened positive for one or more mental disorders, including 29% for PTSD and 33% for MDD (Ricciardelli et al., 2022). These are also lower than estimates from the Canadian general population at the time, where 18% screened positive for one or more mental disorders (Statistics Canada, 2022). These baseline findings suggest CORs enter training with relatively strong mental health, likely reflecting CSC’s pre-employment psychological screening, which is designed to identify and exclude individuals at elevated psychological risk.

### Correctional Officer Recruits, Mental Health, Suicide, and Trauma Exposure

CO work is remarkably complicated due to its physically, socially, emotionally, and psychologically strenuous nature (Siqueira Cassiano & Ricciardelli, 2023). In addition, COs face occupational stressors, both operational and organizational, along with burnout, all found to be associated with adverse outcomes for job performance, health, and personal and family relationships (Lambert et al., 2015; Lambert et al., 2021; Slate & Vogel, 1997).

Although recruits appear psychologically well at occupational entry, research on serving COs consistently reveals significantly elevated prevalence of mental disorders. COs operate in high-stress, high-risk environments characterized by violence, medical emergencies, and exposure to suicide attempts, which are all factors that contribute to elevated risk for PTSD, MDD, panic disorder (PD), alcohol use disorder (AUD), and generalized anxiety disorder (GAD). In a national study of Canadian public safety personnel, CWs reported among the highest prevalence of positive screens: 29.1% for PTSD, 31.1% for MDD, and 23.6% for GAD (Carleton et al., 2018). Provincial data showed an even higher prevalence: in Ontario, 59% of COs screened positive for at least one mental disorder, including 34.2% for PTSD, 39.7% for MDD, and 32.0% for GAD (Carleton et al., 2020), while in Manitoba, 29.9% of COs screened positive for PTSD, 42% for MDD, and 29.5% for GAD (Ricciardelli et al., 2022). Similar trends have been observed internationally, with COs experiencing elevated PTSD, and substance use disorders relative to the general population (Burhanullah et al., 2022; Denhof & Spinaris, 2016; Schultz & Ricciardelli, 2024).

Suicidal thoughts and behaviors are also disproportionately elevated among CWs, including COs (Ricciardelli et al., 2024). In the previously identified study of Manitoba correctional staff, 34.8% reported lifetime suicidal ideation and 15.1% reported ideation in the past year; lifetime suicide planning and attempts were reported by 20% and 8% of respondents, respectively, with past-year planning at 8% and attempts at 2.0% (Ricciardelli et al., 2022). These findings, which showed minimal gender differences, far exceed general population estimates. Furthermore, individuals who screened positive for multiple mental disorders were significantly more likely to endorse suicide-related behaviors, which highlights the psychological toll of correctional work and the widening gap between the psychological health of recruits versus serving officers. These findings point to serious and ongoing risks to psychological well-being within correctional workforces and reinforce the urgency of examining early mental health indicators at the point of occupational entry.

### Trauma Exposure and Mental Health

The elevated prevalence of mental disorders among COs appears closely linked to trauma exposure. Trauma is a well-established risk factor for psychological distress and psychopathology (Kessler et al., 1995; Sareen et al., 2007), and COs report repeated exposure to both acute and cumulative PTEs. Much of the existing literature has emphasized cumulative trauma exposure as a predictor of mental health outcomes. More recent studies of public safety populations suggest certain types of PTE, such as sudden violent death or severe human suffering, may be more strongly associated with psychological symptoms than trauma frequency alone (Carleton et al., 2019). For example, in a study of CWs employed in Manitoba, researchers found that participants who screened positive for PTSD reported higher PTE exposure and greater environmental or occupational stressors at work (McKendy et al., 2023). Similarly, in the United States, researchers found CWs experienced, on average, 28 PTE over the course of their careers and showed higher prevalence of PTSD, MDD, and GAD than the general population, potentially demonstrating a link between compromised mental health and trauma exposure (Spinaris et al., 2012).

The DSM-5 broadened PTSD diagnostic criteria to include not only direct trauma exposure but also “repeated or extreme exposure to aversive details of the traumatic event(s),” such as collecting human remains or police officers repeatedly exposed to accounts of child abuse (American Psychiatric Association, 2013). These criteria are particularly relevant to CWs, who may not always directly witness trauma but are often confronted with its consequences. While not all cumulative exposures meet this revised threshold, this expansion has prompted researchers to investigate whether trauma type, frequency, or their interaction better predicts mental health outcomes in high-risk occupations.

Despite the growing body of research on public safety professionals, COs, especially at occupational entry, have received comparatively limited empirical attention. This is a critical gap, especially in the context of CSC, where psychological screening is integral to the recruitment process, although details around their screening processes and tools are not public knowledge. As CSC (2025) notes,

Pre-employment psychological assessments are reliable and valid predictors of personal suitability and job performance. Their objective is to evaluate specific personality characteristics that will ensure a candidate’s suitability for correctional officer and primary worker/kimisinaw positions. Candidates will need to complete a questionnaire and then a structured clinical interview. This assessment follows the standards of both the Canadian Psychological Association and American Psychological Association. Licensed clinical psychologists conduct the psychological assessments (np).

Understanding the psychological health of recruits before workplace exposure offers a rare opportunity to examine risk and resilience before the onset of occupational trauma.

### This Study

In this study, we ask: *what is the prevalence of mental disorders among COs during training, and once recruited how is their mental health informed by PTE exposures?* Drawing on baseline data from a national sample of CORs, we examine the prevalence of mental disorders and suicidal behaviors before employment. We also assess the presence and characteristics of trauma exposure, whether events are singular or cumulative, whether exposures stemmed from prior professional roles, and whether recruits who screened positive for a mental disorder identified their most distressing trauma as a discrete event or part of an ongoing pattern. These questions aim to inform future research and policy regarding the psychological screening of recruits, the need for trauma-informed training, and the need for longitudinal research to truly interpret the long-term mental health outcomes of correctional careers.

## Method

### Procedure

We analyzed baseline survey data from individuals recruited to CSC who are in the Correctional Training Program (CTP). These CORs (*n* = 792) were invited to complete a baseline survey during CTP between 2018 and 2024^1^. Before arriving at CTP, recruits are invited by email to participate in the survey and reinvited once at CTP.

The survey includes established screening criteria for several mental disorders, suicide behaviors, along with items assessing demographics, history of exposure to PTE, workplaces experiences, personal relationships, and well-being. Respondents provided informed consent before completing the survey and participated voluntarily, with the understanding that their responses had no implications for their employment or relationship with the researchers’ universities.

### Measures

#### Sociodemographic Factors

Participants reported sex (male, female), age group (19–29 years, 30–39 years, 40–49 years, 50–59 years, 60 years, and older), marital status (single, married/common-law, separated/divorced, remarried), education (high school or less, some postsecondary less than 4-year college/university, 4-year college/university or higher), and the province to which they were to be deployed after training (i.e., the federal prisons located in the provinces of Ontario, Manitoba, New Brunswick, Saskatchewan, Nova Scotia, Alberta, British Columbia, and Quebec).

#### Mental Disorder Screens

Positive screens were determined using validated self-report measures and their corresponding cutoff thresholds:

- PTSD: 20-item PTSD Checklist for DSM-5 (PCL-5; past-month symptoms). Total scores range from 0 to 80. Positive screens required exposure to at least one PTE (via the Life Events Checklist for DSM-5), meeting minimum criteria for each PTSD symptom cluster, and a total score >32 (Blevins et al., 2015). Cronbach’s α for the PCL-5 total score was .99.
- MDD: Nine-item Patient Health Questionnaire (PHQ-9; past 14 days). Total scores range 0–27. Positive screen: score >9 (Kroenke et al., 2001). Cronbach’s α for the PHQ-9 total score was .99.
- GAD: Seven-item Generalized Anxiety Disorder Scale (GAD-7; past 14 days). Total scores range 0–21. Positive screen: score >9 (Spitzer et al., 2006). Cronbach’s α for the GAD-7 total score was .98.
- PD: Seven-item PD Severity Scale–Self-Report (PDSS-SR; past 7 days). Total scores range 0–28. Positive screen: score >7 (Shear et al., 1997). Cronbach’s α for the PDSS-SR total score was .98.
- AUD: 10-item AUD Identification Test (AUDIT; past 12 months). Total scores range 0–40. Positive screen: score >15 (Saunders et al., 1993). Cronbach’s α for the AUDIT total score was .99.

A composite variable (“any mental disorder screen”) was computed to indicate whether a participant screened positive on any of the above measures.

#### PTE Exposure

The Life Events Check List for DSM-5 (LEC-5) was used to evaluate participants’ lifetime exposure to 16 types of PTEs (Gray et al., 2004). Participants were classified as having been exposed to a specific PTE if they indicated that (a) the event occurred to them directly; (b) they witnessed the event affecting someone else; (c) they learned about the event happening to a close family member or friend; or (d) they encountered the event as part of their occupational duties as a first responder. A binary variable (yes/no) was generated to denote exposure to any PTE, based on whether the respondent experienced one or more of the 16 events. In addition, the total number of different types of PTE exposures was calculated by summing exposures across all items (total score could range from 0 to 16). Respondents who experienced at least one LEC-5 event were asked to select a single index PTE they perceived as being the worst PTE experience, the most distressing event, or the event that was currently causing the most distress.

#### Suicidal Behavior

Suicidal ideation, planning, and attempts were assessed through a series of yes/no questions: *Ideation*: “Have you ever contemplated suicide?” and “Has this happened in the past 12 months?”, *Planning*: “Have you ever made a serious plan to attempt suicide?” and “Has this happened in the past 12 months?,” and *Attempts*: “Have you ever attempted suicide?” and “Did this happen in the past 12 months?”

### Statistical Analyses

First, we conducted cross-tabulations to investigate how the total number of traumatic exposures (with the chi-square tests), the PTE perceived as the worst or most distressing, and prior occupational experience (with Fisher’s exact tests) were distributed by positive mental disorder screens. Second, to test whether frequency of PTEs, type of PTEs, or their interaction better predicted positive mental disorder screens, we estimated two logistic regression models: Model 1 included only the total number of traumatic exposures, and Model 2 included only the types of trauma. Models were adjusted for suicidal ideation. We also estimated interaction models between total PTE exposures and specific trauma types; however, none of the interaction terms were statistically significant; thus, these results are not reported to maintain clarity and avoid unnecessary complexity.

The dependent variable in all logistic regression models was a binary indicator of any mental disorder screen (0 = *negative screen*; 1 = *positive screen*). The independent variables varied by model. In Model 1, the predictor was the total number of PTE exposures. In Model 2, predictors were specific PTE types and entered as separate binary variables indicating whether the individual had experienced each event type.

## Results

We first describe the demographic characteristics of the sample, followed by prevalence estimates of mental disorders, trauma exposures, and suicide behaviors. We then examine associations between trauma exposure and mental health outcomes, including whether trauma type, frequency, or prior occupational experience predict positive mental disorder screens. Finally, we report results of inferential analyses testing these relationships.

### Demographics

Information was analyzed for 707 (89.3%) respondents (792 respondents logged into the survey, but not all completed it). Demographic information is shown in Table 1. Of those who responded to the sociodemographic questions, 58.6% identified as male and 41.4% identified as female. The average age of all participants was 35.3 years (median = 31.0). Participants reported being married/common-law (51.3%), single (41.8%), separated/divorced (5.1%), or remarried (1.8%). Regarding education, 32.4% completed a 4-year university/college program or higher, 51.9% reported some postsecondary education (less than a 4-year degree), and 15.7% had high school or less. Participants slated to work in Ontario represented the largest group (28.3%), followed by Alberta (18.5%) and British Columbia (16.5%).

**Table 1:** Sample Demographic Information (*N* = 792)

Demographic characteristic	% (*n*)
Sex (*n =* 707)	
Male	58.6 (414)
Female	41.4 (293)
Age (*n* = 695)	
19–29 years	46.0 (320)
30–39 years	31.5 (219)
40–49 years	16.5 (115)
50–59 years	5.6 (39)
60 years and older	0.3 (2)
Marital status (*n* = 682)	
Single	41.8 (285)
Married/Common-law	51.3 (350)
Separated/Divorced	5.1 (35)
Remarried	1.8 (12)
Education (*n* = 663)	
High school or less	15.7 (104)
Some postsecondary	51.9 (344)
4-year university/college program or higher	32.4 (215)
Province (*n* = 672)	
Alberta	18.5 (124)
British Columbia	16.5 (111)
Manitoba	8.3 (56)
New Brunswick	6.1 (41)
Newfoundland and Labrador	0.1 (1)
Nova Scotia	4.0 (27)
Ontario	28.3 (190)
Prince Edward Island	0.3 (2)
Quebec	10.0 (67)
Saskatchewan	7.9 (53)

### Mental Disorder Prevalence and Trauma Exposures

Positive screens for mental disorders are shown in Table 2. A small proportion of CORs screened positive for PTSD (1.6%), MDD (1.6%), GAD (1.8%), PD (0.6%), and AUD (0.4%). Overall, 4.9% screened positive for at least one disorder, and only 0.5% screened positive for three or more disorders. Based on self-report, 7.2% screened positive for any mental disorder, including any mood disorder (2.8%) and any anxiety disorder (3.4%).

**Table 2:** Percentage of Positive Screens for Mental Disorders on Self-report Measures

Mental disorder	% (*n*)	Established cutoff score
Posttraumatic stress disorder (PCL-5), *n* = 556		>32
No	98.4 (547)	
Yes	1.6 (9)	
Depression (PHQ-9), *n* = 585		>9
No	97.8 (572)	
Yes	1.6 (13)	
Generalized anxiety (GAD-7), *n* = 566		>9
No	98.2 (556)	
Yes	1.8 (10)	
Panic disorder (PDSS-SR), *n* = 466		>7
No	99.4 (463)	
Yes	0.6 (3)	
Alcohol use disorder (AUDIT), *n* = 543		>15
No	99.6 (541)	
Yes	0.4 (2)	
Any mood disorder, *n* = 471		
No	97.2 (458)	
Yes	2.8 (13)	
Any anxiety disorder, *n* = 436		
No	96.6 (421)	
Yes	3.4 (15)	
Any mental disorder, *n* = 374		
No	92.8 (347)	
Yes	7.2 (27)	
Total number of mental disorders, *n* = 365		
0	95.1 (347)	
1	2.7 (10)	
2	1.6 (6)	
3 or more	0.5 (2)	

*Note.* PCL-5 = PTSD Checklist for DSM-5; PHQ-9 = Patient Health Questionnaire-9; GAD-7 = Generalized Anxiety Disorder-7; PDSS-SR = Panic Disorder Severity Scale-Self-Report; AUDIT = Alcohol Use Disorders Identification Test.

Figure 1 displays the distribution of lifetime trauma exposures (out of 16 possible PTEs). The mean number of reported traumas was 7.3 (median = 7.0). Figure 2 presents the distribution of PTEs among participants with and without any mental disorder. Among those with a positive screen, the highest prevalence (14.8%) was observed among individuals reporting in the category of 12 to 16 traumatic exposures. However, the chi-square tests indicated no statistically significant difference in the number of exposures between those with and without a positive screen (*p* = .102, χ^2^ = 6.21).

**Figure 1:**
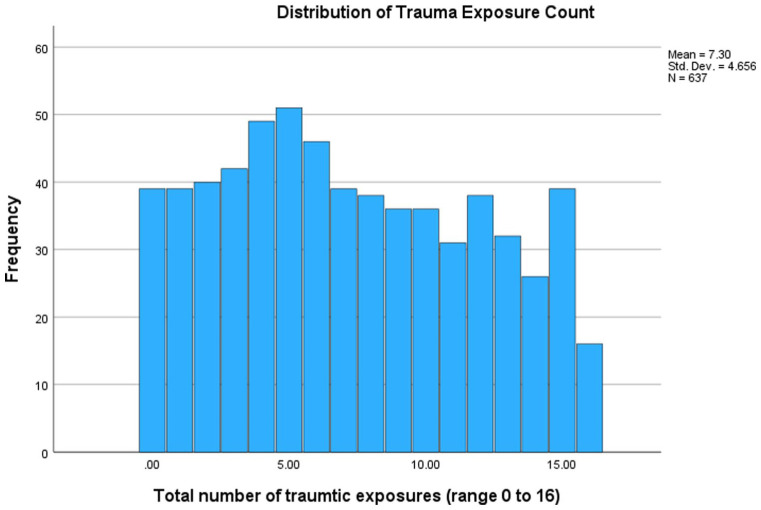
Distribution of the Total Number of Traumatic Exposures

**Figure 2:**
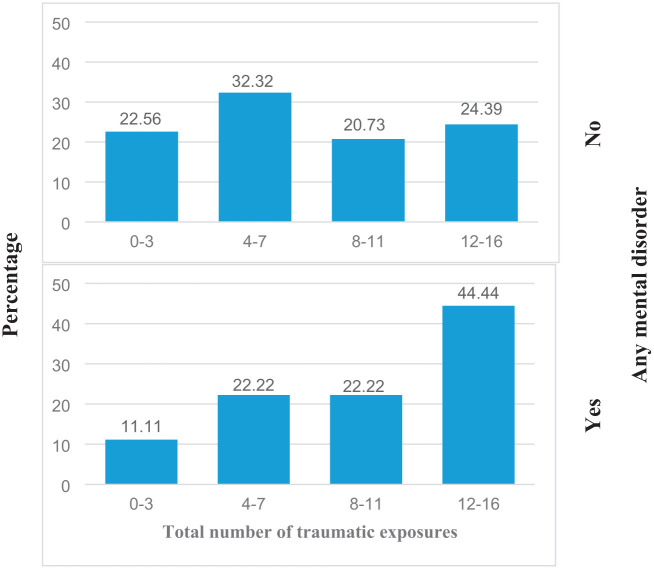
Percentage Distribution of Total Traumatic Exposure Categories Among Participants With and Without Any Mental Disorder

Table 3 summarizes the prevalence of mental disorders by trauma exposure. All 27 participants who screened positive for a mental disorder reported at least one traumatic event, whereas none of the participants without trauma exposure screened positive.

**Table 3:** Prevalence of Mental Disorders Among Participants With and Without Exposure to trauma

Exposure to trauma	Any mental disorder		
	No % (*n*)	Yes % (*n*)	Total % (*n*)
No trauma exposure	100 (3)	0.0 (0)	100 (3)
At least one trauma exposure	92.3 (325)	7.7 (27)	100 (352)
Total	92.4 (328)	7.6 (27)	100 (355)

Table 4 shows the distribution of the traumatic event participants with a positive screen who perceived as the “worst” traumatic event. The most reported index traumas were combat/war zone exposure (25.0%), other very stressful experiences (17.2%), and fire or explosion (16.7%).

**Table 4:** Distribution of Positive Screen for Mental Disorders by Type of Worst Traumatic Event Experienced

Worst traumatic event	Any mental disorder		
	No % (*n*)	Yes % (*n*)	Total % (*n*)
Life-threatening natural disaster	100 (11)	0.0 (0)	100 (11)
Fire or explosion	83.3 (10)	16.7 (2)	100 (12)
Serious transportation accident	97.3 (36)	2.7 (1)	100 (37)
Serious accident at home, work, or during recreational activity	89.5 (17)	10.5 (2)	100 (19)
Exposure to a toxic substance	100 (4)	0.0 (0)	100 (4)
Physical assault	95.1 (39)	4.9 (2)	100 (41)
Assault with a weapon	94.4 (17)	5.6 (1)	100 (18)
Sexual assault	86.4 (19)	13.6 (3)	100 (22)
Other unwanted or uncomfortable sexual activity	86.7 (13)	13.3 (2)	100 (15)
Combat or exposure to a war zone	75.0 (3)	25.0 (1)	100 (4)
Captivity	100 (1)	0.0 (0)	100 (1)
Life-threatening illness or injury	97.1 (33)	2.9 (1)	100 (34)
Exposure to severe human suffering	100 (10)	0.0 (0)	100 (10)
Sudden violent death	92.6 (26)	7.1 (2)	100 (28)
Sudden accidental death	96.4 (27)	3.6 (1)	100 (28)
Serious injury harm or death you caused someone else	100 (4)	0.0 (0)	100 (4)
Any other very stressful event or experience	82.8 (24)	17.2 (5)	100 (29)

### Implications of Prior Public Safety and Military Background

Tables 5 to 7 show associations between prior occupational experience and mental disorder prevalence. Of the 309 participants who responded, 30.1% reported prior experience as Public Safety Professionals (PSP), 21.0% as Correctional Services Personnel (CSP), 31.4% in Health care/Other roles, and 17.5% with Military backgrounds (Table 5). When examining prevalence of positive mental disorder screens across these groups (Table 6), the highest prevalence was observed among Health care/Other Personnel (13.8%), followed by CSP (9.3%), Military (9.1%), and PSP (9.0%). A statistically significant association was observed only for Health care/Other Personnel (Fisher’s exact *p* = .05). Regarding trauma exposure (Table 7), all PSP participants with available data reported experiencing at least one trauma (100.0%, *n* = 84). By comparison, small proportions of CSP (9.1%, *n* = 5), Health care/Other (4.6%, *n* = 4), and Military (2.0%, *n* = 1) participants reported no trauma exposure. The association between trauma exposure and occupational background was statistically significant for PSP (Fisher’s exact *p* = .005) but not for other groups.

**Table 5:** Distribution of Participants’ Prior Occupational Experience

Job categories	% (*n*)
Public Safety Personnel (PSP)	30.1 (93)
Correctional Services Personnel (CSP)	21.0 (65)
Health Care and Other Personnel	31.4 (97)
Military	17.5 (54)
Total	100 (309)

**Table 6:** Distribution of Positive Screen for Mental Disorders by Type of Participants’ Prior Occupational Experience

Job categories	Any mental disorder		Fisher’s exact (*p*)
	No % (*n*)	Yes % (*n*)	
Public Safety Personnel (PSP)	91.0 (61)	9.0 (6)	.60
Correctional Services Personnel (CSP)	90.7 (39)	9.3 (4)	.53
Health Care and Other Personnel	86.2 (50)	13.8 (8)	.05*
Military	90.9 (30)	9.1 (3)	.72

* *p* < .05.

**Table 7:** Distribution of Positive Screen for Trauma total by Type of Participants’ Prior Occupational Experience

Job categories	Trauma		Fisher’s exact (*p*)
	0 trauma % (*n*)	At least one trauma % (*n*)	
Public Safety Personnel (PSP)	0.0 (0)	100.0 (84)	.005*
Correctional Services Personnel (CSP)	9.1 (5)	90.9 (50)	.36
Health Care and Other Personnel	4.6 (4)	95.4 (83)	.80
Military	2.0 (1)	98.0 (48)	.35

* *p* < .05.

### Suicide Behaviors

Table 8 shows the prevalence of lifetime and past-year suicide behaviors. Lifetime suicidal ideation was reported by 11.7% of participants; lifetime suicide planning and attempts were reported by 3.9% and 3.1%, respectively. For past-year suicide behaviors, suicidal ideation was reported by 1.0%, while suicide plans and attempts were each reported by 0.2%.

**Table 8:** Prevalence of Lifetime and Past-year Suicide Behaviors

Suicide behavior	%*(n*)
Lifetime suicide ideation (*n* = 588)	
No	88.3 (519)
Yes	11.7 (69)
Lifetime suicide plan (*n* = 587)	
No	96.1 (564)
Yes	3.9 (23)
Lifetime suicide attempt (*n* = 575)	
No	96.9 (557)
Yes	3.1 (18)
Past-year suicide ideation (*n* = 587)	
No	99.0 (581)
Yes	<1 (<5)
Past-year suicide plan (*n* = 587)	
No	99.8 (586)
Yes	<1 (<5)
Past-year suicide attempt (*n* = 575)	
No	99.8 (574)
Yes	<1 (<5)

*Note.* To protect participant confidentiality, all cell counts <5 are reported as “<5,” and corresponding percentages are rounded to “< 1%.”

### Inferential Analysis

Table 9 presents the results of logistic regression analysis predicting the presence of any mental disorder from PTE exposure.

**Table 9:** Logistic Regression Models Predicting Presence of Any Mental Disorder from Psychologically Traumatic Event (PTE) Exposure

	Any mental disorder	
	OR (95% CI)	AOR (95% CI)
Model 1		
Total number of PTE	1.11* (1.01, 1.21)	1.08 (.98, 1.19)
Model 2		
Life-threatening natural disaster	1.18 (0.53, 2.64)	0.84 (.35, 2.02)
Fire or explosion	1.35 (0.58, 3.11)	1.11 (.46, 2.69)
Serious transportation accident	0.88 (0.38, 2.03)	0.90 (.36, 2.23)
Serious accident at work, home	3.30* (1.11, 9.78)	3.37* (1.08, 10.48)
Exposure to toxic substance	1.99 (0.87, 4.52)	1.93 (.80, 4.66)
Physical assault	5.78 (0.77, 43.42)	4.59 (.59, 35.52)
Assault with a weapon	2.00 (0.85, 4.67)	2.34 (.92, 5.92)
Sexual assault	2.15 (0.90, 5.12)	1.55 (.62, 3.88)
Other unwanted or uncomfortable sexual experience	2.89* (1.13, 7.37)	2.06 (.78, 5.49)
Combat	2.30* (1.02, 5.21)	1.88 (.78, 4.53)
Captivity	1.35 (0.52, 3.51)	0.90 (.32, 2.55)
Life-threatening illness or injury	1.27 (0.57, 2.86)	0.96 (.41, 2.28)
Severe human suffering	2.02 (0.88, 4.64)	1.53 (.63, 3.73)
Sudden violent death	2.58* (1.07, 6.27)	1.91 (.76, 4.84)
Sudden accidental death	2.63* (1.08, 6.38)	2.60* (1.03, 6.60)
Serious injury, harm, or death you caused	0.89 (0.26, 3.07)	1.15 (.31, 4.23)
Any other very stressful event or experience	2.11 (0.82, 5.42)	2.64 (.97, 7.19)

*Note.* AOR = Odds ratio adjusted for suicide ideation.

* *p* < .05.

In Model 1, which assessed the effect of the total number of PTEs, the unadjusted odds ratio (OR) indicated a significant association, with each additional PTE exposure associated with an 11% increase in the odds of screening positive for a mental disorder (OR = 1.11, 95% CI = [1.01, 1.21], *p* = .03). After adjusting for suicidal ideation, the association was attenuated and no longer significant (AOR = 1.08, 95% CI = [0.98, 1.19]). This suggests suicide ideation may partially account for the observed relationship between cumulative PTE exposure and the likelihood of a mental disorder.

In Model 2, specific types of PTEs were examined in relation to the presence of any mental disorder. Experiencing a serious accident at work, home, or during recreational activity was significantly associated with increased odds of a positive mental disorder screen, both before (OR = 3.30, 95% CI = [1.11, 9.78], *p* = .03) and after adjusting for suicide ideation (AOR = 3.37, 95% CI = [1.08, 10.48], *p =*.04). In addition, sudden accidental death remained significantly associated with mental disorder even after adjustment (AOR = 2.60, 95% CI = [1.03, 6.60], *p =*.04). Other exposures, including unwanted sexual experiences, combat, and sudden violent death, were significant in unadjusted models but lost significance after adjustment for suicide ideation.

Across the models, only specific types of PTEs, such as serious accidents at work/home and sudden accidental death, remained significant predictors of mental disorder after controlling for suicide ideation. In contrast, total trauma frequency and its interaction with trauma type were not significant, suggesting that the type of trauma contributes more to positive mental disorder screens than frequency or their combination.

## Discussion

In this study, we examined the mental health at occupational entry of CORs, focusing on mental disorder and suicide behavior prevalence, as well as the relationship between trauma exposure and mental health outcomes. Although some CORs had prior public safety or military backgrounds, only 9.0% screened positive for any mental disorder at baseline. The comparatively low prevalence is perhaps a consequence of CSC’s pre-employment recruit screening practices, which excludes most applicants who have a mental disorder. However, whether these practices support the best long-term outcomes for correctional services remains uncertain. The potential trade-offs of screening out candidates with mental health histories are still not well understood. Longitudinal data, ideally with an appropriate comparison group, will be needed to determine the long-term implications more fully.

Our findings meaningfully advance current knowledge by revealing CSC recruits begin their careers as a psychologically healthy cohort, yet prior research consistently shows serving COs exhibit markedly elevated prevalence of mental disorders compared with the general population (Ricciardelli et al., 2022). The divergence suggests the occupational environment and the work play a central role in the development of compromised mental health over occupational tenure. Organizational, operational, and cultural stressors within the Service and its institutions may, therefore, be central contributors to the decline in wellness among COs. This requires further study with longitudinal data to demonstrate causation.

Notably, baseline psychological health does not imply a lack of PTE exposure at occupational entry. Respondents reported relatively high levels of PTE exposure, with an average exposure to approximately seven distinct trauma types before beginning their CO careers. Given the well-established link between trauma and psychopathology, these findings highlight the value of monitoring mental health over the course of training and early service, as future occupational exposures could interact with prior experiences to influence well-being and mental health. Maintaining recruits’ psychological health, therefore, requires selecting suitable candidates and addressing organizational conditions that place officers at substantial risk for cumulative strain, organizational, operational, and traumatic stress.

Although past-year prevalence of suicidal ideation, planning, and attempts was low, lifetime prevalence was notably higher, with 11.7% reporting suicidal thoughts at some point in their lives. This suggests that some recruits may carry enduring psychological burdens, reinforcing the value of providing consideration to past experiences even when current symptoms are absent or minimal. Our documented prevalence is lower than that found among provincial and territorial servicing CWs (Ricciardelli et al., 2022), although comparisons are complicated because Ricciardelli and colleagues did not assess whether suicide behaviors were due to their work. This further highlights the need for longitudinal data to fully understand how correctional work influences CO mental health and suicide behaviors. Indeed, our findings, contextualized by existing literature, suggest a mental health and suicide crisis in correctional services, at least in part, resulting from the work and emphasize the need for intervention, recognition, policy development, and service transformation.

In addition, our analyses revealed trauma exposure frequency alone did not significantly predict mental health outcomes, after adjusting for suicide ideation. Instead, specific trauma types, particularly serious accidents and sudden accidental death, emerged as stronger and more consistent predictors of positive screens for mental disorders. This suggests that not all PTEs carry equal psychological weight, and that the qualitative nature of trauma may be more important than cumulative exposure. Only a subset of our respondents experienced the specific trauma types most strongly linked to mental disorders, which may explain why the cohort can be both highly trauma-exposed yet largely asymptomatic.

These findings align with emerging evidence in other public safety populations, which suggests that trauma type, rather than sheer number of exposures, may be more predictive of distress (Carleton et al., 2018). Thus, interventions to support mental health after trauma exposure should be tailored and actioned for the exposures most documented to negatively affect CO mental health to be most effective and feasible. While dose–response relationships between trauma exposure and mental disorders have been reported, other work has emphasized the role of contextual and psychological factors, such as perceived life threat, helplessness, or peritraumatic dissociation in shaping outcomes (Brewin et al., 2000; Ozer et al., 2003). Together, this literature supports our observation that specific high-impact trauma types, such as sudden, life-threatening, or high-intensity events, may be more strongly associated with symptom presentation than overall trauma frequency.

We also observed that recruits with prior occupational backgrounds in health care or related fields had the highest prevalence of positive mental disorder screens, while PSPs reported the highest overall trauma exposure. These patterns, while preliminary, may reflect differences in prior job demands, the nature of trauma encountered, or comfort with symptom disclosure. Furthermore, although based on small sample sizes, the traumatic events perceived as the “worst” reported by participants who screened positive for a mental disorder tended to reflect high-intensity or less-specific experiences, such as combat, fire or explosion, or broadly defined “very stressful events.” In some instance, we note, recruits were reluctant to provide specific details or the nature of the event, which limits our knowledge of which events are the most impactful for mental health. Our findings further support the broader evidence that trauma content and perceived salience may play a stronger role in psychological outcomes than cumulative exposure alone and highlight the value of tailoring early supports to the specific histories and PTE profiles of COs. Determining what these supports should look like, however, remains a complex challenge. Little is known about which interventions are effective or likely to be used in this population. Evidence-informed, evaluated solutions are needed—both preventive (e.g., increasing awareness of and ability to recognize mental health changes) and reactively (e.g., interventions for COs experiencing compromised mental health).

Overall, although only a small subset of recruits screened positive for a mental disorder, CORs showed identifiable patterns across trauma type, occupational background, and perceived worst-event descriptions. These findings suggest that early indicators of risk may be detectable even within a highly screened and ostensibly healthy cohort, given certain preservice experiences appear to cluster among those with elevated symptoms. Nevertheless, CORs appeared healthier than both serving COs and the Canadian general population (Carleton et al., 2018; Ricciardelli et al., 2022; Statistics Canada, 2022). Several explanations are possible: CSC’s pre-employment screening likely excludes individuals at higher risk, self-selection may attract candidates with greater baseline resilience, CORs may underreport their responses to appear healthier, and prior exposure to high-stress roles may confer psychological preparedness. Underreporting due to stigma or concerns about employability is also plausible, particularly given recruits are in the hiring process when psychologically assessed by CSC and in our study (Carleton et al., 2020; Wills et al., 2021). However, determining whether underreporting occurs and to what extent is currently unclear and beyond the scope of this study, although it represents an important area for future investigation.

The very fact recruits enter service with a low prevalence of mental disorders, despite meaningful trauma histories have practical implications, particularly given the recruitment and retention challenges facing correctional services in Canada and internationally (Schultz & Ricciardeli, 2024; Siqueira Cassiano & Ricciardelli, 2023). As noted, screening practices that categorically exclude applicants with mental health diagnoses may not be optimally aligned with the realities of correctional work, where the work itself can be psychologically demanding and coping skills and experience with adversity is essential. Evidence of recovery, effective symptom management, or adherence to treatment among applicants who screen positive may be more meaningful indicators of readiness than the absence of symptoms and may better prepare CORs for the demands of prison work. This approach could also reduce stigma by countering assumptions that a past mental health issue inherently predicts future impairment or should deter employability. Thus, services may benefit from refining screening approaches to distinguish between unmanaged symptoms and well-regulated or successfully treated conditions. In addition, given the proximity to the hiring process may shape how comfortable recruits feel disclosing symptoms, services could review how psychological screening procedures and expectations are communicated. Messaging that emphasizes support, confidentiality, and the acceptability of previously treated conditions may reduce fears of negative employment consequences and encourage more accurate reporting. This aligns with broader concerns that strict screening practices may inadvertently encourage underreporting—an important consideration when interpreting the present data. Beyond recruitment, training environments must continue to integrate structured, trauma-informed curriculum that acknowledges the prevalence and relevance of prior PTEs on COR health. Integrating structured psychoeducation, normalizing the range of preservice experiences, and offering early voluntary check-ins may help mitigate stigma and support adjustment during training. Identifying individuals exposed to high-impact trauma types could also inform optional, nonpunitive preventive supports, such as resilience modules, brief clinical consultations, or enhanced peer support, without positioning these recruits as “higher risk.” More broadly, the disconnect between healthy recruits and the elevated prevalence of mental disorders among serving officers reinforces the need for sustained organizational investment in mental health support across the career span. Interventions might include routine psychological screening that is supportive rather than disciplinary, access to confidential care, increased supervisory awareness training, and organizational reforms that address known contributors to deterioration. Our findings provide a baseline foundation upon which institutions can develop more evidence-informed, ethically grounded, and operationally effective strategies for supporting correctional staff well-being.

### Limitations

Our findings are not without limitations. First, the study relied on self-report measures, which may be influenced by underreporting or response bias, particularly in sensitive areas such as mental health and trauma. This could be exacerbated by participants’ proximity to the hiring process, where concerns about employability or stigma may influence disclosure. Future research may benefit from experimentally examining how the framing of psychological screening during recruitment influences symptom reporting among applicants. Second, the current analysis reflects a single time point—baseline during training—limiting conclusions about causality or long-term outcomes. While certain trauma types were associated with elevated symptom scores, if these symptoms persist, worsen, or resolve over time remains unclear. As part of the broader CCWORK protocol, longitudinal diagnostic interviews have also been completed for these recruits, and future analyses will compare this data to examine changes in mental health and relationships to pre- and postrecruitment trauma exposure. Third, some subgroups, particularly those defined by trauma type or prior occupational role, were relatively small, limiting statistical power and generalizability. Fourth, the study did not account for potential protective or moderating variables such as coping style, social support, or access to psychological resources, all of which may shape the relationship between trauma and mental health.

## Conclusion

Our findings emphasize two central points: (1) recruits constitute a psychologically healthy group at baseline, and (2) despite this, they carry substantial trauma histories that vary meaningfully in type and impact. Our data provide an early examination of the relationship between trauma exposure and mental health symptoms in a pre-employment sample of CORs in CSC. Although recruits reported high levels of lifetime trauma exposure, only a small proportion screened positive for a mental disorder. Certain trauma types (e.g., sudden accidental death and serious accidents) were more strongly associated with elevated symptom scores than cumulative exposure.

Overall, we contribute to a growing body of evidence suggesting the nature of trauma exposure may be more relevant than its frequency for understanding early mental health risk. By establishing a pre-employment baseline, this study provides a foundation for future longitudinal research examining how mental health evolves over the course of correctional careers, and how training, workplace supports, and protective factors may shape resilience and vulnerability over time.

## Notes

1. This article differs from prior work using CCWORK data (Easterbrook et al., 2022) in several important ways. Easterbrook et al. relied on an early subsample of baseline respondents (*n* = 265) and focused primarily on demographic characteristics and mental disorder prevalence among correctional recruits. This study analyzes the full CCWORK baseline dataset collected between 2018 and 2024 (*N* = 792 recruits) and provides the first comprehensive examination of recruits’ mental health, suicidal behaviors, and potentially traumatic event exposures at occupational entry, including analyses of trauma type, trauma frequency, and prior occupational experience.
